# The urinary phenolic acid profile varies between younger and older adults after a polyphenol-rich meal despite limited differences in in vitro colonic catabolism

**DOI:** 10.1007/s00394-018-1625-1

**Published:** 2018-02-27

**Authors:** Areej Alkhaldy, Christine A. Edwards, Emilie Combet

**Affiliations:** 1 Human Nutrition, School of Medicine Dentistry and Nursing, College of Medical Veterinary and Life Sciences, University of Glasgow, New Lister Building, Glasgow Royal Infirmary, Alexandra Parade, Glasgow, G31 2ER UK; 2 Clinical Nutrition Department, Faculty of Applied Medical Sciences, King Abdulaziz University, Jeddah, Saudi Arabia

**Keywords:** Ageing, Colon, Metabolism, Microbiota, Polyphenols, Inter-individual variability, Gut, Fermentation, Phenolic acid, Age

## Abstract

**Purpose:**

To investigate whether age influences colonic polyphenol metabolism.

**Methods:**

Healthy participants, younger (*n* = 8; 23–43 years) and older (*n* = 13; 51–76 years), followed a 3-day low-polyphenol diet (LPD) and a 3-day high-polyphenol diet (HPD). Urinary phenolic acids (PA), short chain fatty acids (SCFA), pH and gas were monitored, alongside selected colonic bacteria. Human faecal in vitro fermentations of rutin with or without raftiline were used to evaluate the gut microbiota capacity in a subset of both groups.

**Results:**

Total urinary PA were higher in the older group after HPD compared to the younger group (1.5-fold; *p* = 0.04), with no difference between groups in terms of a change between diets (Δ high-low diet). While 17 PA were detected in all younger participants after HPD, a narrower range (*n* = 8 to 16 PA) was detected in most (*n* = 9/13) older participants, with lower level of benzoic acid (19-fold; *p* = 0.03), vanillic acid (4.5-fold; *p* = 0.04) but higher hippuric acid (2.7-fold; *p* = 0.03). Faecal SCFA concentration did not change after HPD within group, with similar differential excretion (Δ high-low diet) between groups. There were no differences between groups for faecal pH, total, faecal bacteria including *Flavonifractor plautii*, bifidobacteria, and bacteroides. In human in vitro faecal fermentations, seven PAs were detected in both groups after 24 h of rutin fermentation, with no quantitative and modest qualitative differences between groups. Total SCFA in faecal fermentation did not differ between groups, except for butyric acid (twofold higher in the older group; *p* = 0.009) when rutin was fermented with raftiline over 24 h.

**Conclusions:**

Urinary phenolic acids were less diverse in older participants despite limited difference in functional capacity of in vitro faecal fermentations.

**Electronic supplementary material:**

The online version of this article (10.1007/s00394-018-1625-1) contains supplementary material, which is available to authorized users.

## Introduction

The number of adults aged 60 years and over will double between 2000 and 2050, from 11 to 22% [1] globally. The increase in lifespan has a direct effect on the incidence of age-related diseases, including colorectal cancer (CRC), which can put a tremendous strain on health services [2].

The importance of the role of the gut microbiota in health has been increasingly demonstrated. The composition of the colonic microbiota is altered in the elderly with potentially beneficial species, such as *Bifidobacteria, Bacteroides*, and *Lactobacilli* declining, and potentially harmful bacteria, such as *Escherichia coli, Enterobacteriaceae* and *Clostridium perfringens*, increasing [3].

Changes in the colonic microbiota and its metabolic products including short chain fatty acids (SCFA), butyrate, acetate and propionate, could directly affect the proliferation, differentiation, and gene expression of the colonic epithelium [4–6]. Butyrate is the primary energy source for colonocytes, and may play a key role in maintaining homeostasis of the colonic mucosa inhibiting proliferation and inducing apoptosis and differentiation in colorectal cancer cell lines [7, 8].

Plant foods contain a large range of bioactive molecules, including (poly)phenolics. The conversion of the parent (poly)phenolic compound (poorly bioavailable) to smaller phenolic acids (with increased bioavailability) by the colonic microbiota is likely to be an important contributor to the beneficial effect ascribed to the compounds.

There is an emerging body of work on the potential impact of (poly)phenolic metabolites on age-related factors (inflammation, oxidative stress, glycation, dysbiosis) contributing to chronic diseases such as CRC [9–14]. Recently, the International Scientific Association for Probiotics and Prebiotic expanded the definition of the prebiotics to include (poly)phenolics as they are selectively metabolised by gut microbiota and release microbial bioactive molecules (including phenolic acids) that may confer local and systematic health benefits [15]. The interactions between the gut microbiota and (poly)phenols have been described in two ways: (1) some (poly)phenols metabolites have the capability to promote and/or inhibit the growth certain bacteria [16] and (2) the gut microbiota contributes to the production of small potentially bioactive molecules including phenolic acids [17]. (Poly)phenolics may have protective effects in the gastrointestinal tract by: (1) inhibiting the growth of pathogenic species, e.g. *Clostridium spp, Staphylococcus aureus*, and *Bacteroides spp*. [18]; (2) suppressing the adhesion of gut pathogens to human gut cells [19]; (3) enhancing natural killer cell activity and cytokine secretion [20]. In older adults (40–50 years old), a higher urinary concentration of phenolic metabolites of anthocyanin such as syringic acid, *p*-coumaric acid, 4-hydroxybenzoic acid, and homovanillic acid was associated with higher bifidobacteria level (> 4.47 vs. < 1.18 log^10^copies per g faeces) [17].

Research into the bioavailability and metabolism of (poly)phenols, has so far been mostly limited to young adults [21–26], and inter-individual factors likely to impact on the outcome measures have rarely been studied in depth [27–33]. One study, in particular, looked at the effect of ageing on the absorption, metabolism, and excretion of epicatechin in healthy younger and older subjects after the consumption of cocoa flavanols, with little difference observed between subjects; the study did not, however, consider the colonic metabolism of epicatechin, or phenolic acids production [34]. As a result, very little is currently known about the impact of ageing on the metabolic fate of (poly)phenolics in the human gastrointestinal tract, despite the fact that age is a key risk factor for a non-communicable disease development.

As the body ages, several changes may influence the bioavailability and colonic bacterial metabolism of plant (poly)phenolics: (1) longer transit times [35]; (2) increase in conditions such as irritable bowel syndrome, diverticulosis, and colon cancers which are linked with changes in gut microbiota [36–39]; (3) reduced chewing strength, leading to different food choices and lower fibre intake [40]; (4) reduced physical activity which may affect gut function including frequency of bowel movements [41], (5) changes in the composition/diversity of the microbiota [3]. As the majority of (poly)phenolics are metabolised by bacterial enzymes in the colon, this may influence their colonic metabolism.

As there are very limited data on the effect of ageing on the bacterial metabolism of (poly)phenols, this study aimed to test whether age (≥ 50 years) affects the colonic metabolism of dietary (poly)phenolics, with a focus on flavonols, which are ubiquitous in the Western diet. Rutin was used as a “model” flavonol as it is a well-characterised molecule with published evidence related to its breakdown. Rutin is a quercetin glycoside, which resists hydrolysis and is not deglycosylated within the human small intestine by cytosolic β-glucosidase and/or the lactase–phlorizin hydrolase enzymes, and thus pass intact to the large intestine. Rutin is degraded in the colon by the microbiota to low molecular weight, phenolic acids, and is a well-characterised molecule [22, 23]. We hypothesized that the colonic metabolism of (poly)phenols would be less efficient in older adults.

## Methods

### Study design

Two designs were utilised to test the study hypothesis. The first employed a human dietary intervention to compare the colonic metabolism of (poly)phenols between healthy younger (< 45 years) and older (> 50 years) adults, focusing on urinary phenolic acid excretion and gut bacterial composition (focusing on known polyphenol-degrading bacteria). The age cut-off, while arbitrary, was designed to obtain two groups distinct in age. The National Health Service in Scotland uses the age of 50 years as the cut-off for their bowel screening programme, justified by the increased risk of colonic diseases in this age group [42]. The second design employed an in vitro fermentation model, using faecal samples collected during the dietary intervention, to study the metabolic (functional) capacity of the faecal microbiota according to age when a specific flavonol, rutin (quercetin-3-*O*-rutinoside), was fermented.

### Subjects and recruitment

Older (≥ 50 years) and younger (< 45 years) adults were recruited using local advertisements, printed poster displays, and online social networking sites. Exclusion criteria included consuming alcohol (> 4 units/day), obesity (BMI > 30 kg/m^2^), taking dietary supplements, pregnancy or risk of pregnancy, smoking, taking any medication, or having any conditions known to affect bowel function. Approval for this study was obtained from the Ethics committee of the University of Glasgow, College of Medical; Veterinary & Life Sciences (ref FM03110). All participants gave informed written consent.

### Sample size and power calculation

The sample size was calculated with GPower version 3.1 (Dusseldorf university) using urinary phenolic acid excretion as a primary outcome. In younger adults [43], selected urinary phenolic acid excretion increased from 20.6 ± 7.6 to 62.7 ± 44.3 µmol/day after a high-polyphenol diet (∆ high-low diet 42.2 ± 41.0). Pilot analyses showed that excretion varied between ethnic subgroups, with a difference for the ∆ high-low diet of 59 µmol/day between groups (Caucasian ∆ high-low 60.5 ± 36.0 µmol/day; Asian ∆ high-low diet 1.5 ± 10.2 µmol/day; groups with unmatched numbers *d* = 1.6). Assuming a similar effect size of *d* = 1.6, a total of *n* = 16 participants is sufficient to detect (or not) a similar difference (*β* = 80%, two tails, *α* = 0.05). Based on previous experience with similar trials, we allowed for a 40% drop-out rate to recruit a sample of *n* = 13 individuals per group. While recruitment in the older group reached *n* = 13, this was not the case for the younger group (*n* = 8)—as such, the a priori power of the study to detect an effect size of 1.33 is 80% (and 92% for *d* = 1.6) given the sample size and allocation.

### Measurements and sample collection

Anthropometric measurements height, weight, body mass index (BMI), and waist circumference (WC), and blood pressure (BP) were collected at baseline and all subsequent appointments using standard techniques [44]. All participants followed two 3-day diets separated by a 1 week wash-out: a low-polyphenol diet (LPD, avoiding all fruits, vegetables, onions, coffee, tea, chocolate, vanilla and similar flavourings, whole meal products, alcohol, spices, and all dietary supplements) and a high-polyphenol diet (HPD, including flavonoid-rich foods including tomatoes, plums, provided along with cooking guidance and recipes) (Supplementary Table 1). The phenolic composition and fibre content of the HPD are available in Supplementary Table 2. Urine (24 h) was collected on the last day of each diet, and a morning stool sample was collected at the end of the 3-day diet.

### Bowel movements

The usual frequency of bowel movements (twice daily or more, daily, every 2–3 days or less than twice a week) was self-reported retrospectively at the beginning of the study.

### Dietary assessment of (poly)phenol intake

Prospective, weighed dietary records were kept by participants throughout each study period. Diaries were analysed using the WinDiets Nutritional Analysis Software (Robert Gordon University, Aberdeen, UK) [45]. The mean flavonoid content of foods was sourced from the Phenol-Explorer database (http://www.phenol-explorer.eu/contents) [46]. The flavonoid content of low-polyphenol foods such as pasta, bread, biscuits, cakes and pastry was estimated from their wheat flour content [47].

### In vitro fermentation (faecal incubation)

The faecal incubation was prepared as previously described [48]. The substrates used were (a) control (no substrate), (b) 28 µM rutin, and (c) 28 µM rutin (Sigma–Aldrich Company Ltd; Dorset, UK) with 1 g raftiline HP (Orafti, Tienen, Belgium). Raftiline (fibre) was added to the fermentation medium as a source of energy (carbon) to help mimic in vivo conditions (compared to fermentations without a source of carbon or a fast-fermented source such as glucose).

Freshly voided human faecal samples were homogenised with sodium phosphate buffer (0.1 M, pH 7.0) in a blender (Braun™) to make a 32% faecal slurry (16 g faecal sample with 50 ml sodium phosphate buffer). The faecal slurry (5 ml) was added to 44 ml of fermentation medium in 100 ml glass sterilised bottles. The substrate (rutin, 28 µmols) was added to the faecal slurry with or without 1 g of a highly fermentable fibre (raftiline). Control cultures containing no substrates were incubated at the same time. Participants were asked to provide a whole bowel movement, and the same sample was used to produce the faecal slurry added to the four fermentation bottles (a single bottle for each condition). The anaerobic conditions were established by using anaerobic reagents in the media (reducing solution checked with resazurin) and by flushing the media and bottles with oxygen-free nitrogen. The fermentation bottles were sealed with a gas-tight septum, which was fitted with a gas collection syringe monitoring gas production. Fermentation bottles were kept upright in a shaking water bath at 37 °C, 60 strokes/min for 24 h to simulate the colonic lumen conditions. Two samples (3 ml) of fermentation fluid were collected after 0, 2, 4, 6 and 24 h. One sample for each time point was immediately stored at − 80 °C for phenolic acid analysis. The other was mixed with 1M NaOH (1 ml) and stored at − 20 °C for SCFA production measurements.

### Colonic fermentation markers: pH and gas production

A 50-ml disposable syringe and a three-way tap were used to measure gas production in each fermentation bottle at different time points (0, 2, 4, 6, 24 h). An auto-calibrated portable digital pH meter model (Hanna pH20 instruments, USA) was used to measured faecal pH by preparing a suspension of ~ 1 g faecal sample from each participant and diluted in 3 mL of distilled water. The pH of fermentation fluid was determined at 0, 2, 4, 6, and 24 h with universal pH indicator paper from 1 to 14 (Fisher Brand, UK).

### Phenolic acid extraction, derivatization and analysis by gas chromatography mass spectrometry (GC–MS)

Phenolic acids were measured in urine and fermentation fluid. Extraction and derivatisation were carried out as previously described [43]. Derivatized phenolic acids were analysed on a Trace GC interfaced to a DSQ mass spectrometer equipped with a split/splitless injector and an AI3000 autosampler (Thermo Fisher, Hemel Hempstead, UK) as previously described [42]. Identification of phenolic acids was based on retention time (*t*_R_) and target ions [49]. Quantification was based on 2.5 to 15 µg calibration curves of derivatised and analysed phenolic acid standards. The area ratio of each standard was averaged and the coefficient of variance calculated (*R*^2^ > 0.98).

### SCFA analysis by gas chromatography with flame ionization detection (GC-FID)

Short chain fatty acids were measured in dry faeces and fermentation fluid. Extraction and analysis were carried out according to Laurentin and Edwards [50]. SCFAs were estimated using a TRACE™ 2000 gas chromatograph (Thermo Quest Ltd, Manchester, UK) equipped with a flame ionization detector (250 °C) and a Zebron ZB-Wax capillary column (15 m × 0.53 mm id × 1 µm film thickness, catalogue No.7 EK-G007 22, Phenomenex, Cheshire, UK). EK-G007 22, Phenomenex, Cheshire, UK).

### Extraction, concentration, and purity of bacterial DNA

Bacterial DNA was isolated and purified as previously described [51]. DNA concentration and purity were determined by measuring 1.5 µl of undiluted DNA extract with a NanoDrop ND-1000 (software version 3.7.4; Fisher Scientific, UK). An absorbance ratio (A260/A280) greater than 1.8 was used to assess high purity, and the absorbance ratio at 230/260 nm was used to assess guanidinium salt carried. Furthermore, DNA was assessed for shearing by electrophoresis.

### Determination of bacterial diversity and composition

*Flavonifractor plautii* was selected for quantification in faecal samples because of its previous reported contribution to the colonic metabolism of flavonoids [52–54]. Bifidobacteria, bacteroides, and total bacteria were also measured. Real-time PCR with species-specific probes was used to quantify individual species, bacterial populations, and total bacteria (Supplementary Methods) on a 7500 Real-Time PCR System (Applied Biosystems, Carlsbad, CA) using TaqMan. Quantification was performed against serial dilution of bacterial DNA standards obtained from pure cultures (Supplementary Methods). DNA standards for *F. plautii* (DSM 4000; 6.3 ng/µl) were obtained from the German Collection of Microorganisms and Cell Cultures (DSMZ). Standard DNA for bifidobacteria (*Bifidobacterium longum*, DSM 20219T, 9.8 ng/µl), bacteroides [*Bacteroides vulgatus* (DSM 1447T, 27.2 ng/µl)], and total bacteria [*Bacteroides vulgatus* (DSM 1447T, 27.2 ng/µl)] were available in-house [51]. A set of seven bacterial reference standards were prepared for each target. The serial dilution to measure the *Eubacterium ramulus* and *F. plautii* was 1:5, and 1:10 to measure bifidobacteria, bacteroides, and total bacteria.

### Statistical analysis

Data were analysed using Minitab 16. The Anderson–Darling test was used for normality. Descriptive statistics are presented as mean and standard deviation, or medians and inter quartile range (IQR). Comparisons between groups were carried out using Mann–Whitney test for non-parametric data and paired test and two sample *t* tests for normally distributed data. Chi-squared test was used to compare the frequency of bowel movement between groups.

## Results

### Subject characteristics

13 older adults aged between 51 and 76 years old and eight younger adults aged between 23 and 43 years old were recruited, with no subsequent drop-out. The baseline data for participants are presented in Table 1, with no significant differences in anthropometric characteristics between the two groups. Median BMI and WC were within the normal cut-off range for younger and older healthy adults. The male to female ratio was similar between groups, with two males and six females in the younger group and three males and ten females in the older adult group (*p* = 0.99). Diastolic BP was higher (by 17 mmHg) in older adults (*p* = 0.005).

**Table 1:** Baseline data in younger (*n* = 8) and older (*n* = 13) participants

	Younger group (*n* = 8)		Older group (*n* = 13)		*p* value
	Median	IQR	Median	IQR	
Age (years)	23.0	22.0–28.5	61.0	54.0–64.0	0.0002
Height (cm)	163.0	155.3–174.3	161.0	1.6–1.7	0.3
Weight (kg)	63.0	56.4–69.8	63.0	59.4–75.7	0.4
BMI (kg/m^2^)^a^	22.0	20.6–26.4	25.1	23.8–28.4	0.06
WC (cm)^b^	80.0	77.8–80.8	85.0	79.0–98.0	0.1
Systolic BP	122.0	108.5–127.5	120.0	117.0–137.0	0.2
Diastolic BP	69.0	59.8–73.5	86.0	75.0–89.0	0.005

	*n*	%	*n*	%	
Sex (M/F)	2/6	–	3/10	–	0.99*
Normal weight	5	62.5	6	46.1	0.68*
Overweight	3	37.5	7	53.8	–
Obese	0	0	0	0	–

*p value using Fisher’s exact test between age group

^a^BMI cut-off points of overweight [adult = 25, older adult (55–65 years old) = 28]; [55, 56]

^b^WC cut-off points of high risk (adult women = 80 cm, men = 94 cm; older women = 99 cm, men = 106 cm); [55, 56]

Frequency of bowel movements was not different between younger and older groups (*p* = 0.84) with 37.5% in the younger group and 25% in the older group having twice daily or more frequent bowel movement; 37.5% in the younger group and 50% in the older group having daily bowel movement; and 25% in the younger group and 25% in the older group having a bowel movement every 2–3 days.

### Flavonoid intake during low- and high-polyphenol diets (LPD and HPD)

**Fig. 1:**
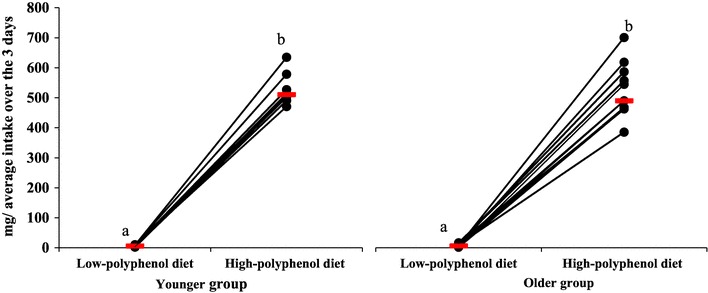
Average flavonoid intake over the 3 days low- and high-polyphenol diets in younger (*n* = 8) and older (*n* = 13) participants. Each circle indicates the estimated average daily flavonoid intake for each participant after low- and high-polyphenol diets. Median flavonoid intake for each group is indicated by a red horizontal line. a, b symbols indicate differences within group (LPD to HPD; *p* = 0.000)

Flavonoid intake during the 3-day LPD was 6 mg/day in the younger (IQR 2–10) and older groups (IQR 2–16). During the 3-day HPD, flavonoid intake in younger adults was 510 mg/day (IQR 499–539) and 496 mg/day (IQR 438–540) in older adults. Flavonoid intake increased from low to high diet for both groups (*p* = 0.000), with no difference between groups (Fig. 1). Moreover, there were no differences between the groups in term of flavonols or phenolic acids intake after the HPD (Supplementary Table 3). As urinary phenolic acid excretion has been shown to be markedly increased between 8 and 24 h following ingestion [31], urinary phenolic acid excretion was corrected for flavonoid intake on day 3 of the diet, given that the 24 h urine collection was carried out from the second urine of day 3, and including the first urine of day 4.

There was no difference between groups for intake on day 3, during the LPD [5 mg (IQR 2–10) versus 9 mg (IQR 5–21) for younger and older groups, respectively] or the HPD [553 mg (IQR 474–666) versus 497 mg (IQR 305–643) for younger and older groups, respectively].

### Dietary fibre intake during low- and high-polyphenol diets (LPD & HPD)

The fibre intake increased after the HPD in both groups (younger: *p* = 0.0009; older: *p* = 0.002) with no difference between groups in terms of change to dietary fibre intake (*p* = 0.6). Dietary fibre intake during the 3-day LPD was 9 g/day (IQR 8–10) in the younger and 12 g/day in the older group (IQR 8–14). During the 3-day HPD, dietary fibre intake in younger adults was 27 g/day (IQR 26–29) and 27 g/day (IQR 24–34) in older adults (Supplementary table 4).

### Macronutrient/ micronutrient intake during low- and high-polyphenol diets (LPD and HPD)

There was no difference between groups in terms of energy, fat, protein, carbohydrate, total sugars, starch, or alcohol intake during either low or HPD, or when considering the difference in macronutrient intake between dietary periods (Δ high-low diet) (Supplementary Table 4). The intake of vitamins or dietary minerals did not differ either, except for thiamine and copper intake during the HPD, with thiamine intake higher (1.3-fold; *p* = 0.05) in the older group, and copper intake higher (1.4-fold; *p* = 0.01) in the younger group (Supplementary Table 5).

### Identification of phenolic acids in urine

A total of 18 urinary phenolic acids were identified and quantified by GC-MS after LPD and HPD in the younger group. However, 4-OHPAA was excluded from the sum of urinary phenolic acid excreted for between-group comparisons, as it is produced by pathways unrelated to the colonic degradation of (poly)phenols [31] and did not increase in young or older adults after the HPD. Subsequently, only *n* = 17 phenolic acids are reported (Table 2).

**Table 2:** Amount of phenolic acid in 24 h urine (µmol/day) after low and high-polyphenol diet in younger (*n* = 8) and older (*n* = 13) participants

Group	Younger								Older								*p* value
Diet	Low-polyphenol diet			High-polyphenol diet			∆		Low-polyphenol diet			High-polyphenol diet			∆		
Phenolic Acid^#^	*N*	Median	IQR (*Q*1 to *Q*3)	*N*	Median	IQR (*Q*1 to *Q*3)	Median	IQR (*Q*1 to *Q*3)	*N*	Median	IQR (*Q*1 to *Q*3)	*N*	Median	IQR (*Q*1 to *Q*3)	Median	IQR (*Q*1 to *Q*3)	
BA [23]	8/8	16.6†	10.8 to 25.1	8/8	31.1§	13.9 to 46.8	13.0	4.7 to 24.0	13/13	1.1†	0.8 to 2.3	13/13	1.8§	1.1 to 5.4	0.5	0.0 to 4.0	0.03
PAA [23]	8/8	3.2†	1.7 to 4.0	8/8	3.2	0.0 to 4.8	0.6	− 1.7 to 1.3	7/13	0.9†	0.6 to 1.0	6/13	1.1	0.6 to 1.3	0.2	0.1 to 0.4	0.80
MA [23]	8/8	0.7	0.4 to 0.9	8/8	1.1	0.5 to 1.7	0.6	0.0 to 0.8	13/13	0.3	0.3 to 0.8	13/13	0.5	0.5 to 1.0	0.2	0.0 to 0.2	0.14
3-OHBA [23]	8/8	0.6*	0.2 to 0.7	8/8	0.9*	0.3 to 1.5	0.3	0.2 to 0.7	8/13	0.4	0.2 to 1.3	9/13	1.2	0.3 to 1.5	0.1	0.0 to 0.5	0.27
3-OHPAA [22, 24, 57]	8/8	2.3*	1.9 to 3.2	8/8	19.2*	10.7 to 34.0	16.6	8.8 to 27.2	13/13	2.3**	1.6 to 3.1	13/13	30.2**	18.7 to 57.8	26.3	17.1 to 54.7	0.40
4-OHBA [23]	8/8	2.1	1.5 to 2.5	8/8	3.8	2.5 to 4.4	1.3	0.2 to 1.9	12/13	2.2	1.6 to 3.7	12/13	2.9	1.7 to 3.8	0.1	− 0.4 to 0.7	0.17
4-OHPPA [23, 57]	8/8	0.4	0.1 to 0.4	8/8	0.5	0.1 to 1.0	0.2	00 to 0.6	8/13	0.2	0.1 to 0.6	8/13	0.3	0.1 to 0.6	1.4	− 14.1 to 8.8	0.08
VA [23, 57]	8/8	0.7*	0.4 to 0.8	8/8	2.8*	1.3 to 4.1	2.0	0.6 to 3.4	9/13	0.9	0.2 to 1.3	12/13	1.4	1.1 to 2.3	0.9	0.3 to 2.3	0.04
HVA [22, 23, 57]	8/8	10.8*	7.4 to 11.8	8/8	19.8*	10.7 to 23.4	9.5	4.8 to 15.2	13/13	7.6**	6.0 to 7.9	4/13	12.4**	11.7 to 17.3	6.9	4.1 to 9.4	0.71
4-OHMA [57]	8/8	4.9	4.5 to 6.0	8/8	3.9	3.1 to 4.7	− 0.5	−2.4 to − 0.2	13/13	3.7**	3.0 to 4.0	13/13	2.6**	2.0 to 3.1	− 1.1	− 1.2 to 0.2	0.84
3,4diOHBA [23]	8/8	1.0*	0.9 to 1.1	8/8	2.1*	1.6 to 2.7	1.1	0.7 to 1.5	4/13	0.9	0.9 to 1.0	4/13	1.2	1.0 to 1.4	0.5	0.4 to 0.6	0.20
3,4diOHPAA [22, 23]	8/8	1.5*	1.3 to 1.7	8/8	2.9*	2.1 to 7.4	2.0	0.6 to 5.5	13/13	1.2**	0.9 to 1.3	13/13	3.2**	2.2 to 6.0	2.0	1.4 to 5.1	0.91
HA [23, 57]	8/8	155.7*	104.8 to 200.5	8/8	969.8*§	633.9 to 1174.1	856.7	502.0 to 987.4	13/13	301.5**	153.7 to 431.4	13/13	1734.5**§	937.4 to 2641.9	1507.8	653.2 to 2500.9	0.06
Dihydrocaffeic acid [23]	8/8	1.3*	0.8 to 1.7	8/8	2.3*	1.3 to 3.4	1.0	0.5 to 1.4	6/13	1.3	1.0 to 1.4	9/13	1.5	1.2 to 2.3	0.8	0.3 to 1.0	0.54
3,4diOHPPA [23]	8/8	0.5*	0.2 to 0.6	8/8	1.6*	0.7 to 1.8	0.9	0.6 to 1.4	8/13	0.3**	0.1 to 0.7	9/13	0.8**	1.7 to 1.1	0.4	0.2 to 0.8	0.07
GA [23]	8/8	0.6	0.2 to 0.9	8/8	0.8	0.3 to 1.7	0.5	0.2 to 0.8	4/13	0.8	0.6 to 1.1	5/13	0.7	0.5 to 1.0	0.3	− 0.2 to 0.4	0.25
3-OHhippA [22, 57]	8/8	21.0*	17.6 to 27.8	8/8	68.6*	41.7 to 83.1	40.9	19.0 to 55.2	13/13	15.1**	10.8 to 21.6	13/13	28.4**	24.3 to 44.3	15.1	7.2 to 20.8	0.76
Total		205.2	158.1 to 251.8		1196.7	729.7 to 1344.7	911.3	517.3 to 1074.5		336.2	190.2 to 468.5		1819.1	986.1 to 2831.1	1562.5	696.6 to 2641.7	0.06

*N* number of participants; *BA* benzoic acid; *PAA* phenylacetic acid; *MA* mandelic acid; *3-OHBA* 3-hydroxybenzoic acid; *3-OHPAA* 3-hydroxyphenylacetic acid; *4-OHBA* 4-hydroxybenzoic acid; *4-OHPAA* 4-hydroxyphenylacetic acid; *4-OHPPA* 4-hydroxyphenylpropionic acid; *VA* vanillic acid; *HVA* homovanillic acid; *4-OHMA* 4-hydroxymandelic acid; *3,4diOHBA* 3,4-dihydroxybenzoic acid; *3,4diOHPAA* 3,4-dihydroxyphenylacetic acid; *HA* hippuric acid; *Dihydrocaffeic acid* 3-(3,4-dihydroxyphenyl) propionic acid; *3,4diOHPPA* 4-hydroxy-3-methoxy-phenylpropionic acid; *GA* gallic acid; *3-OHhippA* 3-hydroxyhippuric acid

#Phenolic acids previously described in the literature. ∆ Difference in urinary excretion (high diet minus low diet). *p* value is the difference in urinary phenolic acid excretion (∆high–low diet) between groups. *Significant increase in younger group after the high-polyphenol diet *p* ≤ 0.01. ** Significant increase in older group after the high-polyphenol diet *p* ≤ 0.01. §Significant difference between groups after the low-polyphenol diet *p* ≤ 0.05. † Significant difference between groups after the high-polyphenol diet *p* ≤ 0.05

### Urinary phenolic acid excretion after low- and high-polyphenol diets (LPD and HPD)

**Fig. 2:**
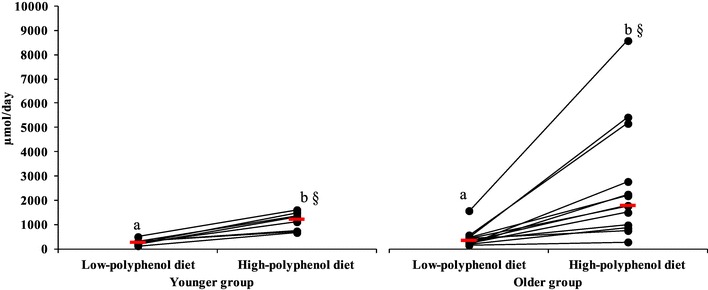
24-hour urinary phenolic acid profile excretion (µmol/day) after low and high-polyphenol diets in younger (*n* = 8) and older (*n* = 13) participants. Each circle indicates the measurement of the urinary phenolic acids profile for each participant after low and high-polyphenol diets. Median urinary phenolic acids for each group are indicated by a red horizontal line. a, b symbols indicate differences within group (LPD to HPD; younger: *p* = 0.0009; older: *p* = 0.0002). §Symbol indicates differences between groups (HPD vs. HPD; *p* = 0.04)

The sum of the 17 urinary phenolic acids excreted increased  ~ 5.6-folds in the younger group from 205 µmol/day (IQR 158–525) after the LPD to 1197 µmol/day (IQR 730–1345) after the HPD (*p* = 0.0009), and ~ 5.5-fold in the older group, from 336 µmol/day (IQR 190–469) to 1819 µmol/day (IQR 986–2831) respectively (*p* = 0.0002). The change in urinary excretion (Δ high-low diet) was not different between groups; however, the urinary phenolic acid excretion was higher after the HPD in the older group (*p* = 0.04) compared to the younger group (Fig. 2), with a large variability in urinary PA excretion in the older group. Correcting for flavonoid intake did not reveal further difference in differential excretion between the groups. Based on the key (statistical) differences seen between LPD and HPD, the main phenolic acids linked to rutin metabolism that are excreted in urine, by at least threefold, are 3-OHPAA, HVA, 3,4DOHPAA, 3,4 DIOHPPA, and 3-OHhippA.

Hippuric acid (HA) was always the most abundant acid in urine samples in both groups (80 and 98% of total phenolic acids for younger and older groups, respectively), and was higher in the older group after the HPD (2.7-fold; *p* = 0.03) compared to LPD. However, the change in excretion, Δ high-low diet, was not different between groups.

**Fig. 3:**
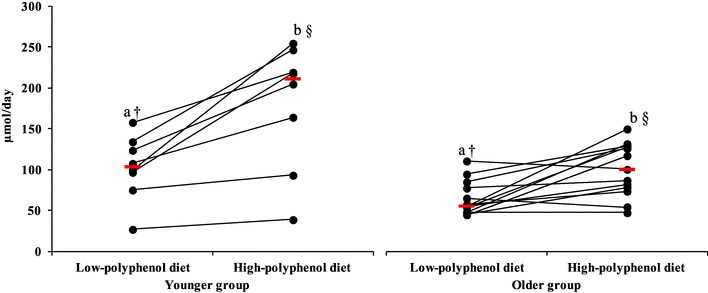
24-hour urinary phenolic acid profile excretion without hippuric acid (µmol/day) after low- and high-polyphenol diets in younger (*n* = 8) and older (*n* = 13) participants. Each circle indicates the measurement of the urinary phenolic acids profile without hippuric acid for each participant after low- and high-polyphenol diets. Median urinary phenolic acids profile without hippuric acid for each group is indicated by a red horizontal line. a,b symbols indicate differences within group (LPD to HPD; younger: *p* = 0.003; older: *p* = 0.007). †Symbol indicates differences between groups (LPD vs. LPD; *p* = 0.03). §Symbol indicates differences between groups (HPD vs. HPD; *p* = 0.02)

The sum of urinary phenolic acids minus HA was considered (as HA is most likely to be formed in the liver by conjugation of benzoic acid and glycine, rather than from flavonoid metabolism). The sum of PA excreted after the HPD increased in both groups (compared to LPD), ~ twofold in the younger group, from 104 µmol/day (IQR 91–129) to 211 µmol/day (IQR 146–233) (*p* = 0.003) and in the older group, from 56 µmol/day (IQR 48–77) to 100 µmol/day (IQR 78–127) (*p* = 0.007). The difference in urinary excretion (Δ high-low diet) remained similar between groups. However, urinary phenolic acid concentration was higher after the LPD (*p* = 0.03) and HPD in the younger group (*p* = 0.02); Fig. 3.

Most notably, there were important qualitative and quantitative differences between groups for the excretion of individual phenolic acids (∆ high-low diet). While 17 phenolic acids were identified in all younger participants after both diets, only eight phenolic acids were excreted by all older participants (BA, MA, 3-OHPAA, HVA, 4-OHMA, 3,4diOHPAA, HA, 3-OHhippA) (Table 2). A narrower range of urinary phenolic acids (*n* = 8–16) was detected in most (*n* = 9/13) older participants. The older group excreted 19-fold less BA (*p* = 0.03), and 4.5-fold less VA (*p* = 0.04) than the younger group (Table 2).

### Faecal pH after low- and high-polyphenol diets (LPD and HPD)

After the HPD, the faecal pH significantly decreased in the older group from 7.7 (IQR 7.4–7.9) to 6.9 (IQR 6.7–7.4; *p* = 0.006), but not in the younger group (7.2, IQR 6.9–7.5 to 6.7, IQR 6.4–7.1). Looking at pH changes with diet (Δ high-low diet for pH), there was no significant difference between the groups (Supplementary Fig. 1).

### Faecal SCFA after the low- and high-polyphenol diets (LPD and HPD)

Total faecal SCFA concentration (sum of all SCFA) did not change significantly from low to HPD in either group despite a threefold increase in fibre intake [younger from 164 µmoles/g dwt (IQR 124–218) to 192 µmoles/g dwt (IQR 162–214); older from 258 µmoles/g dwt (IQR 168–280) to 265 µmoles/g dwt (IQR 208–301)]. Although there were no significant differences (Δ high-low diet) between the groups, the mean SCFA concentration was 1.4 fold higher in the older group compared to the younger group after the HPD (*p* = 0.01; Supplementary Fig. 2). There were no differences in the changes (Δ high-low diet) in each specific acid between groups. However, the absolute levels of acetic acid were higher after both the LPD (1.5-fold; *p* = 0.01) and HPD (1.4-fold; *p* = 0.02) in the older group, compared to the younger group. Absolute levels of heptanoic acid were higher after the HPD in the younger group (1.8-fold; *p* = 0.03; Supplementary Table 6).

### Concentration of bacterial DNA isolated from faecal samples after LPD and characteristics of the qPCR run condition

High quality and high yield DNA was obtained from all faecal samples. The purity and yield of the extracted DNA was high (1.7–2.0 absorbance ratio 280/260 nm; yield 463 ng/µL (IQR 429–552) for older group samples, 519 ng/µL (IQR 480–576) for the younger group samples). The faecal DNA appeared intact and compact as a high-molecular-weight band when electrophoresed through a 1.5% agarose gel. The qPCR amplification efficiency was within the normal range (90–105%) for the total bacteria, *Bacteroides–Prevotella*, and *Flavonifractor plautii* in both groups; however, efficiency for *Bifidobacterium spp*. was just below the normal range (87%) in both groups. The coefficient of determination (*R*^2^) range was between 0.994 and 0.999 for all bacterial groups and species in both groups. Absolute levels of bifidobacteria, bacteroides, and *Flavonifractor plautii* did not differ between younger and older groups (Table 3).

**Table 3:** Absolute (log10/g) faecal concentrations and relative abundance of bacterial groups/species in younger (*n* = 8) and older (*n* = 12) participants

Target	Dry weight sample^a^			
	Younger (*n* = 8)		Older (*n* = 12)	
Log^10^/g	Median	IQR	Median	IQR
Total bacteria	11.9	11.7–12.0	11.8	11.4–11.9
*Bifidobacterium* spp.	10.1	9.7–10.5	10.1	9.2–10.2
*Bacteroides* + *Prevotella*	11.0	10.2–11.2	10.7	10.4–10.7
*Flavonifractor plautii*	8.9	8.4–9.2	8.1	7.7–8.5
%	30 (28–44)		39 (35–47)	

% of faecal water				
*Bifidobacterium* spp.	1.6		2.0	
*Bacteroides* + *Prevotella*	12.6		7.9	
*Flavonifractor plautii*	0.1		0.0	

^a^Calculated using the weight (g) and the moisture percentage (%) of the wet faecal samples

### In vitro fermentation of rutin

Ten fermentations were carried out using the stool samples of six younger (one male and five females) and four older subjects (one male and three females), collected after the LPD. Rutin was fermented for 24 h with or without fibre (raftiline) to test the metabolic capacity of the gut faecal contents, including the microbiota, in relation to ageing. There was no change in the pH of the fermented faecal fluids containing rutin alone, over time, in either group (Supplementary Fig. 3). However, raftiline and the combination of rutin + raftiline reduced pH from 7 to 5 by 24 h, in both groups. There was no difference in total gas production between groups after 24 h. In both younger and older groups, the combination of rutin + raftiline increased gas production more than rutin alone (*p* = 0.02; *p* = 0.03; respectively). Moreover, the older group produced 1.9-fold more gas over 24 h fermentation than the younger group when raftiline was fermented alone (*p* = 0.02; Supplementary Table 7).

### Metabolism of rutin in faecal fluids and phenolic acids formation

Seven phenolic acids were detected in fermentations of rutin with faecal samples from younger and older adults, over 24 h, with great variability within and between groups (Table 4).

**Table 4:** Accumulation of seven phenolic acids (µmol/L) after 0, 6, and 24 h of fermentation in faecal fluids from younger (*n* = 6) and older (*n* = 4) groups

Phenolic acid^a^	Group	Substrates	0 h	6 h			24 h	
			Median	IQR (*Q*1−*Q*3)	Median	IQR (*Q*1−*Q*3)	Median	IQR (*Q*1−*Q*3)
PAA [59]	Younger	Blank	3.7 (*n* = 6/6)	3.0–4.0	15.2 (*n* = 6/6)	6.0–28.1	66.1 (*n* = 6/6)	46.8–72.2
		Raftiline	2.7 (*n* = 6/6)	2.9–5.2	8.6 (*n* = 6/6)	7.4–8.8	6.6 (*n* = 6/6)	6.9–10.6
		Rutin	3.3 (*n* = 6/6)	2.3–3.9	6.0 (*n* = 6/6)	3.5–11.3	81.9 (*n* = 6/6)	44.4–95.5
		Rutin + raftiline	4.3 (*n* = 6/6)	2.5–3.2	8.6 (*n* = 6/6)	5.3–9.8	8.6 (*n* = 6/6)	3.9–10.1
	Older	Blank	6.9 (*n* = 4/4)	6.2–7.7	92.8 (*n* = 4/4)	38.7–138.2	88.8 (*n* = 4/4)	81.4–101.4
		Raftiline	7.0 (*n* = 4/4)	4.4–9.5	22.2 (*n* = 4/4)	15.8–42.1	26.6 (*n* = 4/4)	18.4–44.7
		Rutin	6.7 (*n* = 4/4)	4.2–8.8	58.7 (*n* = 4/4)	12.7–110.8	128.3 (*n* = 4/4)	93.4–147.3
		Rutin + raftiline	6.4 (*n* = 4/4)	3.8–8.6	17.5 (*n* = 4/4)	9.1–42.0	25.3 (*n* = 4/4)	18.9–40.8
3-OHPAA [48, 57, 59]	Younger	Blank	0.2 (*n* = 1/6)	–	0.2 (*n* = 1/6)	–	0.5 (*n* = 2/6)	0.4–0.6
		Raftiline	0.3 (*n* = 1/6)	–	0.2 (*n* = 1/6)	–	0.1 (*n* = 1/6)	–
		Rutin	0.2 (*n* = 1/6)	–	1.5 (*n* = 2/6)	1.1-2.0	4.4 (*n* = 5/6)	10.4–24.3
		Rutin + raftiline	0.1 (*n* = 1/6)	–	1.3 (*n* = 1/6)	–	1.3 (*n* = 2/6)	0.9–1.7
	Older	Blank	ND	–	0.1 (n = 1/4)	–	0.1 (*n* = 1/4)	–
		Raftiline	ND	–	ND	–	ND	–
		Rutin	0.1 (*n* = 1/4)	–	0.8 (*n* = 2/4)	0.5–1.1	4.2 (*n* = 3/4)	3.2–29.8
		Rutin + raftiline	ND	–	0.3 (*n* = 3/4)	0.2–1.2	0.6 (*n* = 3/4)	0.4–0.8
4-OHBA [48, 57]	Younger	Blank	ND	–	ND	–	ND	–
		Raftiline	ND	–	0.1 (*n* = 1/6)	–	0.1 (*n* = 1/6)	–
		Rutin	0.1 (*n* = 1/6)	–	0.1 (*n* = 1/6)	–	0.1 (*n* = 1/6)	–
		Rutin + raftiline	ND	–	0.1 (*n* = 1/6)	–	0.1 (*n* = 1/6)	–
	Older	Blank	0.2 (*n* = 2/4)	0.2–0.2	0.4 (*n* = 3/4)	0.3–12.2	0.3 (*n* = 3/4)	0.2–13.8
		Raftiline	0.2 (*n* = 1/4)	–	0.2 (*n* = 1/4)	–	0.1 (*n* = 1/4)	–
		Rutin	0.3 (*n* = 2/4)	0.1–0.3	0.2 (*n* = 2/4)	0.1–0.2	0.2 (*n* = 2/4)	0.1–0.2
		Rutin + raftiline	0.2 (*n* = 2/4)	0.1–0.2	0.3 (*n* = 2/4)	0.2–0.2	0.4 (*n* = 2/4)	0.3–0.4
3-OHPPA [27, 60]	Younger	Blank	1.6 (*n* = 2/6)	1.2–2.0	2.8 (*n* = 3/6)	1.6–3.0	2.9 (*n* = 3/6)	1.7–3.3
		Raftiline	0.5 (*n* = 3/6)	0.4–1.8	0.9 (*n* = 5/6)	0.7–1.2	1.3 (*n* = 5/6)	1.2–2.6
		Rutin	0.4 (*n* = 4/6)	0.3–1.1	1.8 (*n* = 5/6)	1.1–1.9	4.8 (*n* = 6/6)	2.0–10.0
		Rutin + raftiline	0.4 (*n* = 3/6)	0.4–1.3	1.6 (*n* = 4/6)	0.5–5.0	1.2 (*n* = 5/6)	0.8–3.4
	Older	Blank	0.9 (*n* = 4/4)	0.6–1.7	4.2 (*n* = 4/4)	0.6–8.2	3.4 (*n* = 4/4)	2.2–4.9
		Raftiline	1.0 (*n* = 4/4)	0.6–2.9	2.8 (*n* = 4/4)	1.0–5.0	3.4 (*n* = 4/4)	2.7–4.1
		Rutin	0.5 (*n* = 4/4)	0.4–1.3	7.7 (*n* = 4/4)	1.6–25.1	13.4 (*n* = 4/4)	11.7–20.1
		Rutin + raftiline	1.3 (*n* = 4/4)	0.5–2.3	9.5 (*n* = 4/4)	1.6–22.4	9.4 (*n* = 4/4)	3.0-18.9
4-OHPPA [57]	Younger	Blank	0.4 (*n* = 2/6)	0.3–0.6	6.5 (*n* = 4/6)	3.6–10.3	1.2 (*n* = 4/6)	0.8–3.4
		Raftiline	0.6 (*n* = 2/6)	0.4–0.6	3.8 (*n* = 4/6)	2.9–4.1	3.9 (*n* = 4/6)	3.1-4.0
		Rutin	0.5 (*n* = 2/6)	0.3–0.6	1.1 (*n* = 4/6)	0.9–1.7	4.9 (*n* = 5/6)	1.6–7.7
		Rutin + raftiline	0.5 (*n* = 1/6)	–	1.0 (*n* = 3/6)	0.9–2.2	1.2 (*n* = 3/6)	1.0–2.0
	Older	Blank	0.4 (*n* = 3/4)	0.2–0.8	3.4 (*n* = 3/4)	1.8–14.6	21.6 (*n* = 4/4)	10.3–37.6
		Raftiline	0.3 (*n* = 3/4)	0.2–0.9	1.4 (n = 3/4)	0.9–1.4	2.0 (*n* = 3/4)	1.3-2.0
		Rutin	0.8 (*n* = 4/4)	0.3–1.3	1.6 (*n* = 4/4)	1.3–2.1	6.9 (*n* = 4/4)	5.0-14.2
		Rutin + raftiline	0.3 (*n* = 3/4)	0.2–0.9	0.4 (*n* = 3/4)	0.4–0.7	1.9 (*n* = 3/4)	1.4–4.6
3,4diOHPAA [48, 59]	Younger	Blank	ND	–	ND	–	ND	–
		Raftiline	ND	–	ND	–	ND	–
		Rutin	ND	–	16.1 (*n* = 6/6)	8.7–35.9	32.9 (*n* = 6/6)	5.5–62.4
		Rutin + raftiline	0.2 (*n* = 1/6)	–	5.0 (*n* = 6/6)	1.7–32.1	7.1 (*n* = 6/6)	2.9–32.9
	Older	Blank	ND	–	ND	–	ND	–
		Raftiline	ND	–	ND	–	3.2 (*n* = 1/4)	–
		Rutin	0.3 (*n* = 1/4)	–	63.0 (*n* = 3/4)	32.0–66.3	65.8 (*n* = 3/4)	35.4–79.1
		Rutin + raftiline	0.5 (*n* = 1/4)	–	48.3 (*n* = 3/4)	24.8–48.7	52.0 (*n* = 3/4)	27.1–53.0
3,4diOHPPA [59]	Younger	Blank	ND	–	ND	–	ND	–
		Raftiline	ND	–	ND	–	ND	–
		Rutin	ND	–	0.2 (*n* = 1/6)	–	0.4 (*n* = 2/6)	0.4–0.5
		Rutin + raftiline	Nd	–	ND	–	ND	–
	Older	Blank	Nd	–	ND	–	ND	–
		Raftiline	Nd	–	ND	–	ND	–
		Rutin	0.2 (*n* = 1/4)	–	1.1 (*n* = 2/4)	0.5–1.6	0.5 (*n* = 2/4)	0.4–0.4
		Rutin + raftiline	ND	–	ND	–	0.6 (*n* = 1/4)	–

*PAA* phenylacetic acid; *3-OHPAA* 3-hydroxyphenylacetic acid; *3-OHPPA* 3-hydroxyphenylpropionic acid; *4-OHBA* 4-hydroxybenzoic acid; *4-OHPPA* 4-hydroxyphenylpropionic acid; *3,4diOHPAA* 3, 4-dihydroxyphenylacetic acid; *3,4diOHPPA* 4-hydroxy-3-methoxy-phenylpropionic acid; *ND* not detected

^a^Phenolic acids previously described in the literature

Only PAA and 3-OHPPA were detected in all fermentations, while 3-OHPAA (younger: 5/6; older 3/4) and 4-OHPPA (younger: 5/6; older 4/4) were detected in most fermentations. 3,4diOHPAA, a recognised rutin metabolite, was seen in all younger donors and most older donors (younger: 6/6; older 3/4). Finally, 4-OHBA (younger: 1/6; older 2/4) and 3,4diOHPPA (younger: 2/6; older 2/4) were detected in the fermentations of some donors only. Five of these phenolic acids were also detected in the control fermentation at 0 h: PAA was detected in all younger and older donors; however, 4-OHPPA (younger: 2/6; older 3/4), 3-OHPPA (younger: 2/6; older 4/4), 4-OHBA (older only 2/4), and 3-OHPAA (younger only 1/6) were detected in the fermentations of some donors (Table 4).

**Fig. 4:**
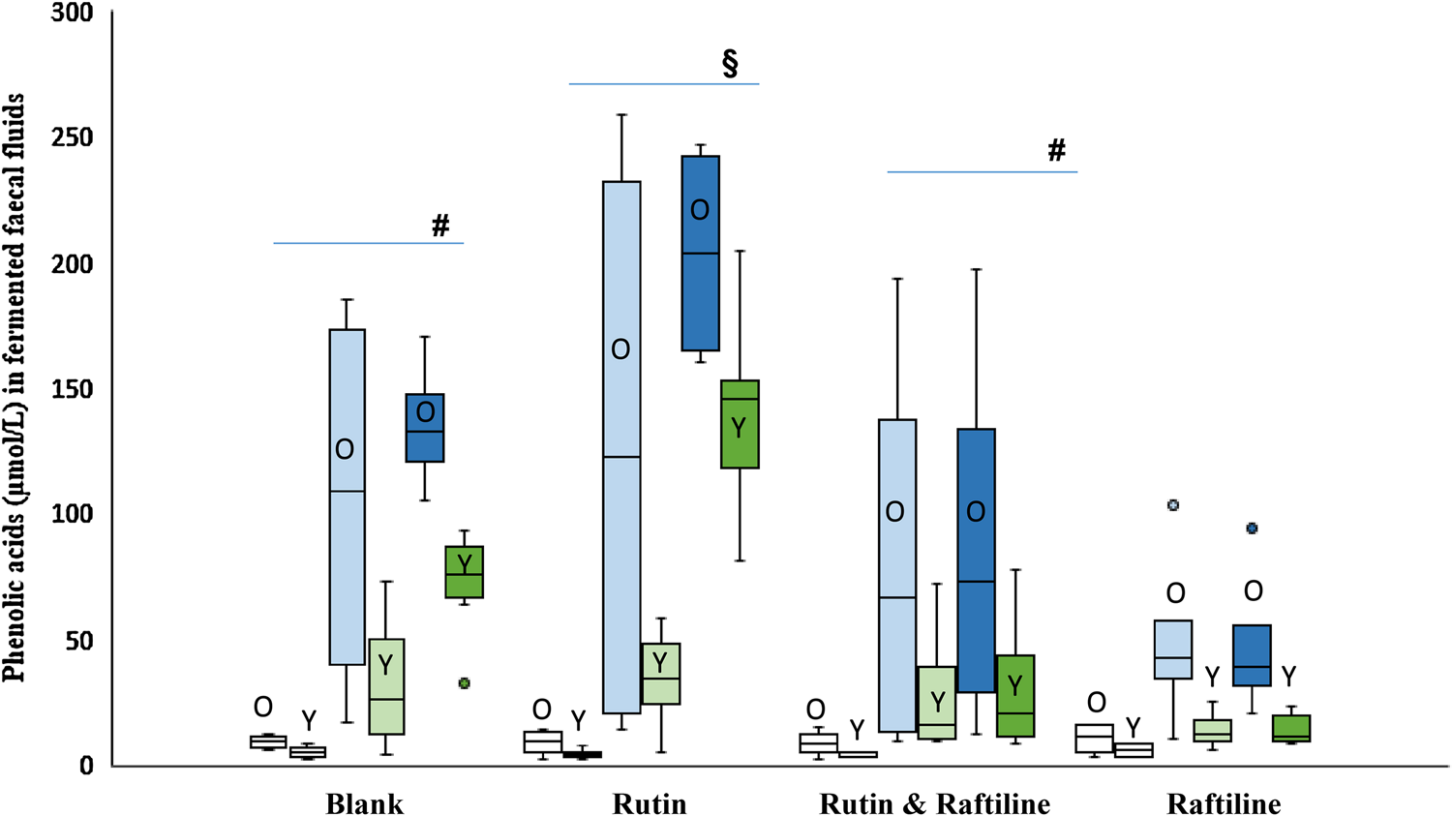
Sum of all (*n* = 7) phenolic acids (µmol/L) in fermented faecal fluids from older (*n* = 4) and younger (*n* = 6) adults (over 24 h of fermentation). The white boxes represent * t* = 0 h, the light shaded boxes  * t*= 6 h and the dark shaded boxes,* t* = 24 h. Boxes representing the older group are labelled “O”, boxes representing the younger group are labelled “Y”. #There was a statistically significant increase in the sum of PA in younger adults over time in the blank fermentations (*p* < 0.001) and when rutin and raftiline were combined (*p* = 0.001). § There was a statistically significant increase in the sum of PA in older adults over time in the fermentations with rutin alone (*p* = 0.04)

The sum of the seven phenolic acids increased over time in all rutin fermentations, with or without raftiline in the younger group (35-fold with rutin only, *p* = 0.000, sixfold with rutin + raftiline, *p* = 0.001) and in the older group when rutin was fermented alone (21-fold, *p* = 0.04), without significant increase with the addition of raftiline (ninefold change, *p* = 0.06). There was no difference in the sum of the seven acids between groups after 24 h in all rutin fermentations, with or without raftiline. The addition of raftiline to the fermentation inhibited the formation of all seven phenolic acids after 24 h, by sevenfold (*p* = 0.02) in the younger group and 2.8-fold (*p* = 0.33) in the older group with no detectable difference between groups (Fig. 4).

### Individual phenolic acids formed after the fermentation of rutin

PAA increased over time in the fermented faecal fluids from the younger group with rutin (25-fold; *p* = 0.000) and rutin + raftiline (2.1-fold; *p* = 0.03), and in the fermented faecal fluids from the older group with rutin only (19-fold; p = 0.04) but not with the addition of raftline. The older group formed more PAA in the fermentations of rutin + raftiline (threefold; *p* = 0.01) and raftiline only (fourfold; *p* = 0.03) compared with the younger group but no differences were detected between groups when rutin alone was fermented. 3-OHPPA also increased in the fermented faecal fluids containing rutin only in both younger (13-fold, *p* = 0.02) and older (25-fold, *p* = 0.03) groups, with no differences between the groups (Table 4).

### Metabolism of rutin in and SCFA production faecal incubation

Total SCFA concentrations increased over time in the fermentations of rutin + raftiline (38-fold for the younger group, *p* = 0.005; 58-fold for the older group, *p* = 0.03) and rutin only (13-fold for younger group, *p* = 0.005; 14-fold for the older group, *p* = 0.03) with no differences between groups. Acetic and propionic acid did not differ between the groups but butyric acid concentration was higher in the older group (two-fold;* p* = 0.009) at 24 h (Supplementary Table 8).

## Discussion

This study tested the hypothesis that age influences the metabolism of dietary (poly)phenols, which may be relevant for gut health and the development of chronic diseases. To our knowledge, this is the first dietary semi-controlled study investigating the colonic metabolism of dietary polyphenols in different age groups using human feeding and *in vitro* faecal fermentation designs. 3 days on each diet was enough for (poly)phenol-rich foods to be supplied to the colon and be fermented over the course of several meals. The present study showed:

1. Inter and intra variability in younger and older groups in terms of urinary excretion of phenolic acids after LPD/HPD.
2. Limited differences between groups in phenolic acid formation after fermentation of rutin with in vitro batch faecal incubations.

Previous work compared absorption, metabolism, and excretion of epicatechin in healthy younger and older subjects, with limited differences in flavonol metabolites levels in plasma and 24-h urinary collection between the age groups [34]. Our own results align with these findings, showing limited quantitative differences between groups in term of phenolic acid excretion, as catabolites of (poly)phenolics or colonic metabolism capacity. These same findings, however, highlight important qualitative differences between the two age groups. While all younger participants excreted all 17 phenolic acids in urine after the 3-day high-polyphenols diet, not all older participants excreted the full panel. This varied from one participant excreting 8 out of 17 phenolic acids to another excreting 17 out of 17 phenolic acids. This is particularly relevant as inter-individual variability in the response to (poly)phenolics (including flavonols) is now being increasingly considered [61]. However, whether inter-individual variability of (poly)phenols metabolism suggests a biological difference between the age groups is still unclear.

The phenolic acids consistently seen in urines from both younger and old subjects were BA, MA, 3-OHPAA, HVA, 4-OHMA, 3,4diOHPAA, HA, and 3-OHhippA with 3-OHPAA, HVA, and 3,4diOHPAA, well described rutin metabolites [27, 48]. Intermediates (3,4-diOHBA, 3,4-diOHPAA, 3,4-diOHPPA) were detected in variable amounts at different time points, but the absence of these phenolic acids at any given point does not simply illustrate capacity, but also the kinetics in flavonol catabolism. The evidence in this study and that of others is clearest 3-OHPAA and 3,4-diOHPAA, as they are both found in the in vivo and in vitro studies. Three more, HVA, 3,4-diOHPPA, and 3-OHhippA were present in the in vivo study after the HPD in both age groups, but not in the in vitro fermentations—highlighting the likely role of phase II metabolism in the liver in the synthesis of these compounds. However, VA, GA, 4-OHMA, 3,4-diOHBA which were present in all younger participants, but only in some of the older participants, could be linked to age-related variations.

With their simpler structure and increased bioavailability, phenolic acids are one of the major (poly)phenol metabolites present in blood with a maximum plasma concentration of 0–4 µmol/L with an intake of 50 mg aglycone equivalents [62], and reported anti-inflammatory, anti-carcinogenic, anti-proliferative, anti-glycative and prebiotic properties in in vitro and/or in vivo studies [16, 62–65]. As the concentration in the colon is much higher than in the circulation with concentration ranging from 46 to 479 µmol/L in human faecal water [59, 66], some effects exerted at high doses may still be physiological [67]. Moreover, while phenolic acid intake is usually in the range of 50–900 mg per day, phenolic acids in urines can reach levels close to 1 mmol/day—as urinary phenolic acid largely come from the colonic metabolism of (poly)phenolics, but also other compounds such as benzoic acid and precursors (quinic acid, aromatic amino acid tryptophan, tyrosine, and phenylalanine). Other sources of benzoic acid are benzoates (E numbers 210–219) which are commonly used in food, medications, and mouthwashes [68, 69].

The bioactivity of specific phenolic acids is less well defined, with examples including 3,4diOHPAA an inhibitor of enzymes involved in detoxification (GSTT2), inflammation (COX-2) and anti-proliferative activity in HCT-116 colon cancer cells [67, 70]; 3,4-diOHPPA (3 µM) and 3,4-diOHPAA (3 µM), inhibitors of the pro-inflammatory cytokines such as TNF-a, IL-1b and IL-6 in lipopolysaccharide-stimulated peripheral blood mononuclear cells [70, 71]; and gallic acid (882 µM) inhibitor of *Clostridium histolyticum* in in vitro faecal fermentation [65, 72].

The intra-group variation we observed could be due to the variation in gut microbiota, themselves influenced by genetics, lifestyle, diet and ageing. Older adults excreted a higher amount of hippuric acid in their urine. Hippuric acid is formed in the liver by conjugation of colonic benzoic acids with glycine; therefore, the high amount of HA could come from sources other than (poly)phenol-rich food metabolism, such as amino acids [68, 69]. Moreover, urinary hippuric acid correlated with hypertension and obesity [73, 74], which would be consistent with the characteristics of some in our older group. The high intra-group variability within the older group for the sum of the phenolic acids was mainly attributable for hippuric acid (30-fold difference between low and high excreters); however, the variability reduced to only threefold when hippuric acid was not included in the sum of phenolic acids.

Several potential mechanisms could explain the absence of some of the phenolic acids in the urine of participants as well as the low amount of phenolic acid excreted in the urine of the older group.

The first mechanism involves a decrease in colonic absorption. As the individual ages, colonic absorption is reduced and the mucosal surface area is diminished [75, 76]. During in vitro faecal fermentation of rutin, a high amount of PAA was detected in the faeces-only control at 0 h in the older group, suggesting that bacterial phenolic metabolism is not the sole source of increased PAA. The comparatively greater formation of PAA in the fermentation fluid of the older participants in the presence of rutin may also point toward a higher capacity of faecal material from older participants to effectively metabolise rutin with the low urinary phenolic acid excretion due to poorer absorption of the phenolic compounds in the colon. Variability in enterohepatic (re)circulation is not well studied and could be an additional contributing factor [77].

The second mechanism relates to the effect of ageing on the colonic microbiota composition and associated microbial and enzymatic activities. In the human colon, bacterial enzymes (β-glucosidases, β-glucuronidases, and α-rhamnosidase) hydrolyse rutin to release the quercetin aglycone [78]. Insufficient or lower levels of bacterial enzymes in the colon could be one reason for the absence and low urinary phenolic acid in the older group. Although these bacterial enzymes were not measured directly in this present study, we could not detect a difference between groups in the quantity of bifidobacteria, bacteroides, or *Flavonifractor plautii* bacteria. The aim of the current protocol focusing on targeted bacteria was not to study the impact of dietary components, but instead to establish how pre-HPD levels of these bacteria could factor in the differences in phenolic acid excretion. Selected bacteria were measured in representative faecal samples and the values were not corrected for the total amount of daily faecal output, which could affect the accuracy of the measurement. Indeed, as the bacteria measurements were performed after the low-polyphenol diet which was lower (but not devoid of) in fibre (younger: 9 g/day; older: 12 g/day), with a view to describe the levels of the targeted bacteria before exposure to the HPD. It is not clear how fibre intake impacts, short term (here, 3 days) on bacterial diversity and total numbers. Additionally, the selected bacteria measured in this study may not be representative of all bacteria responsible for (poly)phenol metabolism found in the colon such as *Enterococcus casseliflavus* [53, 79] *Butyrivibrio* spp. [80], and *Bacteroides distasonis* [58], and may have been influenced by the nutrients in the low-polyphenolic diet (although the impact of such a short-term intervention on bacterial populations themselves is not clear). Based on this study’s finding, the differences in colonic fermentation between groups, as modeled in vitro, are unlikely to be linked to the differences seen in the urinary phenolic acid excretion between groups. Woodmansey et al. [81] reported that a high faecal pH in the elderly (due to a low fibre intake) may lead to a reduction in SCFA production. In the present study, faecal pH was close to 8.0 in the older group after the LPD and above 7.0 after the HPD. However, even though the SCFA were higher in the older group after the HPD, there were no differences between groups when considering the change from the low to high-polyphenol diet. As mentioned above, the measurement of the SCFA was performed in a representative faecal sample and correction for the total daily faecal output was not possible. A pH above 7.0 could be due to other compounds such as ammonia, which have been reported in older adults [81], either due to lower fibre intake and/or increased activity of proteolytic bacterial species, such as Fusobacteria, Propionibacteria, and Clostridia.

It is important when studying the bioavailability of bioactive molecules to consider stratification of the population into responders and non-responders. Age and other factors such as gender, genetics, gut microbiota or physiological status involved in creating inter-individual variability are important to consider before developing such stratification models. Improvement in the knowledge of factors such as age, gender, genetic and gut microbiota composition, and their influence on the metabolism of plant food bioactive molecules, together with the development of methods to identify responsiveness profiles will enhance the development of effective and innovative products for prevention of chronic disease [82]. The strength of this study includes the combination of in vivo and in vitro designs to assess the colonic bacterial metabolic ability of each group. However, the full profile of (poly)phenol metabolites was not measured in the urine and the phenolic acids were not measured in faecal samples, which could have provided useful information regarding the absorption and accumulation of the phenolic compounds in the colon. Only selected bacterial species were measured in the faecal samples, and other gut bacteria such as, *Clostridium scindens, Eubacterium desmolans, Eubacterium ramulus* [52, 60, 79, 83], *Butyrivibrio sp* [80], and *Bacteroides distasonis* [58] could potentially contribute to the colonic metabolism of dietary (poly)phenols.

The differential excretion of phenolic acids in older adults may be linked to differences in the gut microbiota profile or functionality, but may also be linked to other gastrointestinal factors. Age was shown to impact on the phenolic acid profiles excreted in urine between groups qualitatively, as well as quantitatively (the younger group excreted higher phenolic acid in urine after both low- and high-polyphenolic diets). These differences were not explained by the *in vitro* fermentations, as a similar range of phenolic acids was formed from the substrates in both groups, with minimal quantitative differences. Our study highlights the importance of study participant selection (and description) in experimental design aiming to explore the colonic metabolism of plant bioactives. Age may be a factor influencing the considerable variance in measurements seen in some studies. The restricted urinary phenolic acid profile observed in some participants in the older group may have relevance for colonic health, and should be investigated further.

## Electronic supplementary material

Below is the link to the electronic supplementary material.

Supplementary material 1 (DOCX 165 KB)
