# Supplementary material for: The urinary phenolic acid profile varies between younger and older adults after a polyphenol-rich meal despite limited differences in in vitro colonic catabolism
Source: Eur J Nutr. 2018 Feb 27;58(3):1095–111. doi: 10.1007/s00394-018-1625-1 (PMC6499760; doi:10.1007/s00394-018-1625-1)
Supplement: Supplementary file 1 — Supplementary material 1 (DOCX 165 KB) [file 394_2018_1625_MOESM1_ESM.docx]

**The urinary phenolic acid profile varies between younger and older adults after a polyphenol-rich meal despite limited differences in in-vitro colonic catabolism.**

Areej Alkhaldy^1,2^, Christine A. Edwards^1^, Emilie Combet^1^

^1^Human Nutrition, School of Medicine Dentistry and Nursing, University of Glasgow, Glasgow, United Kingdom

^2^Clinical Nutrition Department, Faculty of Applied Medical Sciences, King Abdulaziz University, Jeddah, Saudi Arabia

**Corresponding author**: Dr Emilie Combet, Human Nutrition, School of Medicine, Dentistry and Nursing, College of Medical Veterinary and Life Sciences, University of Glasgow, New Lister Building, Glasgow Royal Infirmary, Alexandra Parade, Glasgow G31 2ER

Email: Emilie.combetaspray@glasgow.ac.uk

Tel 0044 141 201 8527

**Online Supplemental Tables and Figures**

**Supplementary Table 1: Food items provided as part of the high polyphenols diet (HPD - 3 days)**

|  | **Day 1** | **Day 2** | **Day 3** |
| --- | --- | --- | --- |
| **Breakfast** | 1 plum | 1 plum | 1 plum |
|  | 1 glass of purple grape juice | 1 glass of purple grape juice | 1 glass of purple grape juice |
|  | 1 cup of black tea | 1 cup of black tea | 1 cup of black tea |
|  |  |  |  |
| **Snack** | One line of dark chocolate (85%) | One line of dark chocolate (85%) | One line of dark chocolate (85%) |
|  | 1 cup of black tea | 1 cup of black tea | 1 cup of black tea |
|  |  |  |  |
| **Lunch** | 5 Cherry tomatoes | 5 Cherry tomatoes | 9 Cherry tomato |
|  | 1 can of tomato soup | 1 can of tomato soup | 1 can of fresh onion soup |
|  | 1 plum | 1 plum | 1 plum |
| **Snack** | 1 cup of black tea | 1 cup of black tea | 1 cup of black tea |
|  | One line of dark chocolate (85%) | One line of dark chocolate (85%) | One line of dark chocolate (85%) |
|  |  |  |  |
| **Dinner** | 20 g Sun dried tomatoes (~5) | 20 g Sun dried tomatoes (~5) | 20 g Sun dried tomatoes (~5) |
|  | 5 black olives | 5 black olives | 5 black olives |
|  | Pasta and tomato sauce | Chicken Balti ready meal | Rice & tomato sauce |
|  | (see recipe) | 1/3 punnet of raspberry | 1/3 punnet of raspberry |
|  | 1/3 punnet of raspberry | Glass of purple grape juice | Glass of purple grapes juice |
|  | Glass of Purple grape juice |  |  |
|  |  |  |  |
| **Snack** | Cup of black tea | Cup of black tea | Cup of black tea |
|  | One line of dark chocolate (85%) | One line of dark chocolate (85%) | One line of dark chocolate (85%) |

**Supplementary Table 2: Estimated (poly)phenol and dietary fiber available from the standard HPD diet (provided foods only)**

|  | **Day 1** | **Day 2** | **Day 3** | **Total (3 days)** | **Average** |
| --- | --- | --- | --- | --- | --- |
| **Anthocyanins (mg)** | 183.5 | 183.5 | 183.5 | 550.5 | 183.49 |
| **Catechins (mg)** | 53.4 | 53.4 | 53.4 | 160.1 | 53.38 |
| **Flavanols (mg)** | 0.0 | 0.0 | 0.0 | 0.0 | 0.00 |
| **Flavanones (mg)** | 2.0 | 2.0 | 2.0 | 6.1 | 2.03 |
| **Flavones (mg)** | 2.3 | 1.6 | 1.6 | 5.6 | 1.87 |
| **Flavonols (mg)** | 51.8 | 36.4 | 88.2 | 176.3 | 58.77 |
| **Theaflavins (mg)** | 23.6 | 23.6 | 23.6 | 70.9 | 23.65 |
| **Flavonoids (total) (mg)** | 461.2 | 445.1 | 496.9 | 1403.2 | 467.73 |
| **Lignans (mg)** | 0.1 | 0.1 | 0.1 | 0.4 | 0.1 |
| **Ellagitannins (mg)** | 29.3 | 29.3 | 29.3 | 87.8 | 29.7 |
| **Hydroxybenzoic acid dimers (mg)** | 3.8 | 3.8 | 3.8 | 11.4 | 3.8 |
| **Hydroxybenzoic acids (mg)** | 68.9 | 67.8 | 67.8 | 204.5 | 68.2 |
| **Hydroxycinnamic acids (mg)** | 21.4 | 20.0 | 20.0 | 61.5 | 20.5 |
| **Hydroxyphenylacetic acids (mg)** | 0.6 | 0.5 | 0.5 | 1.6 | 0.5 |
| **Hydroxyphenylpropanoic acids (mg)** | 0.9 | 0.6 | 0.6 | 2.2 | 0.7 |
| **Methoxybenzoic acids (mg)** | 8.5 | 6.3 | 6.3 | 21.0 | 7.0 |
| **Methoxycinnamic acids (mg)** | 12.9 | 11.1 | 11.1 | 34.7 | 11.6 |
| **Methoxyphenylacetic acids (mg)** | 8.1 | 6.0 | 6.0 | 20.0 | 6.7 |
| **Phenolic acids (mg)** | 121.3 | 112.3 | 112.3 | 345.9 | 115.3 |
| **Dietary fiber (g)** | 13.5 | 14.5 | 14.5 | 42.5 | 14.2 |

Supplementary Table 3: (Poly)phenolic intake (in mg/3 days) after the high-polyphenol diet in younger (n=8) and older (n=13) estimated using dietary records.

|  | **Younger** | | **Older** | | **p-value*** |
| --- | --- | --- | --- | --- | --- |
| **FAMILY_NAME** | **Median** | **IQR** | **Median** | **IQR** |  |
| Anthocyanins | 198.7 | 185.3-212.5 | 195.1 | 178.7-204.3 |  |
| Catechins | 53.2 | 45.7-53.2 | 40.0 | 30.7-53.4 |  |
| Flavanols | 0.7 | 0.0-1.2 | 1.4 | 0.0-6.9 |  |
| Flavanones | 4.9 | 4.9-6.0 | 3.6 | 2.8-3.8 |  |
| Flavones | 3.2 | 2.8-3.3 | 2.3 | 1.7-3.0 |  |
| **Flavonols** | **95.9** | **88.4-100.2** | **86.6** | **69.5-96.0** | **0.15** |
| Theaflavins | 23.6 | 19.7-23.6 | 17.7 | 13.3-23.6 |  |
| **Flavonoids (total)** | **510.4** | **499.5-539.6** | **496.8** | **438.1-540.3** | **0.32** |
| Lignans | 0.2 | 0.2-0.2 | 0.1 | 0.1-0.2 |  |
| Ellagitannins | 30.1 | 29.2-31.5 | 27.8 | 19.5-29.3 |  |
| Hydroxybenzoic acid dimers | 3.9 | 3.8-4.1 | 3.6 | 2.5-3.8 |  |
| Hydroxybenzoic acids | 70.4 | 67.1-71.1 | 64.4 | 42.5-70.5 |  |
| Hydroxycinnamic acids | 29.4 | 23.9-163.4 | 199.5 | 148.6-549.7 |  |
| Hydroxyphenylacetic acids | 0.8 | 0.7-0.9 | 0.6 | 0.4-0.8 |  |
| Hydroxyphenylpropanoic acids | 1.1 | 1.0-1.2 | 0.8 | 0.6-1.1 |  |
| Methoxybenzoic acids | 11.0 | 9.8-11.5 | 7.7 | 5.8-10.6 |  |
| Methoxycinnamic acids | 14.6 | 13.3-14.9 | 12.1 | 9.6-13.7 |  |
| Methoxyphenylacetic acids | 10.5 | 9.3-11.0 | 7.3 | 5.5-10.2 |  |
| **Total phenolic acids** | **147.2** | **124.7-272.3** | **298.1** | **206.7-648.8** | **0.18** |

P values are calculated between groups, for the most relevant (poly)phenolic groups.

**Supplementary Table 4: Estimated energy and macronutrient intake from the dietary records after low and high-polyphenol diets in younger (n=8) and older (n=13) participants.**

|  | **Low polyphenol diet** | | | | **Low polyphenol diet** | | | |
| --- | --- | --- | --- | --- | --- | --- | --- | --- |
| **Nutrient** | **Younger** | | **Older** | | **Younger** | | **Older** | |
|  | **Median** | **IQR**  (Q1-Q3) | **Median** | **IQR**  (Q1-Q3) | **Median** | **IQR**  (Q1-Q3) | **Median** | **IQR**  (Q1-Q3) |
| **Energy (KJ)** | 8016 | 6729-9195 | 7768 | 6510-8145 | 8227 | 6728-9171 | 7531 | 6166.3-8640.3 |
| **kcal (Kc)** | 1910 | 1609-2194 | 1874 | 1554-1939 | 1949 | 1585-2171 | 1794 | 1451.3-2069.0 |
| **Fat (g)** | 95.8 | 84-111 | 82.1 | 65.6-96.6 | 74.3 | 64-90 | 63.8 | 53.7-85.4 |
| **protein (g)** | 80.4 | 75-82 | 94.3 | 66.3-96.0 | 54.9 | 480-63.6 | 47.1 | 42.1-64.4 |
| **Carbohydrate (g)** | 195 | 124-207 | 195.4 | 161.3-230.5 | 247.1 | 203.9-313.4 | 217.5 | 190.9-269.6 |
| **Total sugars (g)** | 36.1 | 30-57 | 34.3 | 21.3-63.4 | 115.9 | 105.2-155.2 | 92.9 | 79.6-121.7 |
| **Starch (g)** | 140.5 | 97-160 | 149.5 | 122.9-162.7 | 70.6 | 50.6-94.7 | 60 | 49.1-84.8 |
| **Alcohol (g)** | 0 | 0 | 0 | 0 | 0 | 0 | 0 | 0.0-0.5 |
| **Dietary fibre* (g)** | 9.1 | 8 - 10 | 11.8 | 8.0-13.7 | 27.5 | 26.1-29.3 | 26.5 | 23.9-33.8 |

There was no statistically significant difference between older and younger groups

**Supplementary Table 5: Estimated micronutrient intake from the dietary records after low and high-polyphenol diets in younger (n=8) and older (n=13) participants.**

| **Diet** | **Low-polyphenol diet** | | | | | **High-polyphenol diet** | | | | |
| --- | --- | --- | --- | --- | --- | --- | --- | --- | --- | --- |
| **Group** | **Younger** | | **Older** | | **p value** | **Younger** | | **Older** | |  |
| **Nutrient** | **Median** | **IQR**  (Q1-Q3) | **Median** | **IQR**  (Q1-Q3) |  | **Median** | **IQR**  (Q1-Q3) | **Median** | **IQR**  (Q1-Q3) | **p value** |
| **Vitamin A (µg)** | 632 | 563.6-765.2 | 574.0 | 375.3-697.3 | 0.8 | 362.2 | 286.6-384.7 | 383.3 | 271.7-652.0 | 0.4 |
| **Thiamine (mg)** | 1.2 | 1.0-1.3 | 1.1 | 0.9-1.3 | 0.6 | 0.6 | 0.4-0.7 | 0.8 | 0.6-0.9 | 0.05 |
| **Riboflavin (mg)** | 1.7 | 1.5-1.9 | 1.2 | 1.1-1.9 | 0.4 | 0.6 | 0.5-0.9 | 0.9 | 0.8-1.1 | 0.2 |
| **Niacin (mg)** | 28.6 | 26.2-31.0 | 35.9 | 27.0-42.7 | 0.2 | 19.5 | 15.1-29.9 | 16.2 | 12.5-22.0 | 0.6 |
| **Vitamin B6 (mg)** | 1.5 | 1.4-1.6 | 1.4 | 1.0-1.8 | 0.6 | 0.7 | 0.7-1.1 | 0.9 | 0.5-1.3 | 0.5 |
| **Vitamin B12 (µg)** | 5.2 | 4.7-6.0 | 3.7 | 1.9-11.7 | 0.9 | 1.6 | 0.5-2.6 | 2.0 | 0.7-3.1 | 0.6 |
| **Folic acid (µg)** | 155.2 | 141.0-211.5 | 137.0 | 117.0-159.0 | 0.4 | 95.5 | 85.8-119.1 | 125.7 | 116.7-149.0 | 0.2 |
| **Pantothenic acid (mg)** | 4.6 | 3.6-5.1 | 5.5 | 3.2-6.9 | 0.3 | 2.2 | 1.9-2.5 | 3.2 | 1.9-3.5 | 0.2 |
| **Biotin (µg)** | 26.0 | 24.2-30.1 | 30.9 | 17.8-37.8 | 1.0 | 18.5 | 15.2-21.6 | 22.8 | 18.2-25.3 | 0.3 |
| **Vitamin C (mg)** | 17.2 | 14.2-22.6 | 22.0 | 5.7-25.4 | 0.5 | 62.4 | 55.1-66.1 | 75.6 | 38.2-143.1 | 0.6 |
| **Vitamin D (µg)** | 2.0 | 1.3-2.6 | 3.4 | 1.0-5.1 | 0.3 | 1.2 | 0.6-1.7 | 0.8 | 0.3-1.3 | 0.3 |
| **Vitamin E (mg)** | 5.8 | 4.3-8.1 | 5.4 | 2.7-7.0 | 0.6 | 11.7 | 9.6-14.6 | 7.8 | 7.1-11.0 | 0.1 |
| **Calcium (mg)** | 1302.3 | 984.3-1523.8 | 909.7 | 695.0-1069.7 | 0.5 | 358.8 | 333.3-402.9 | 517.0 | 454.7-678.3 | 0.08 |
| **Magnesium (mg)** | 182.7 | 148.1-213.9 | 172.3 | 141.7-223.7 | 0.9 | 146.5 | 128.9-184.0 | 158.3 | 140.0-222.7 | 0.7 |
| **Sodium (mg)** | 3009.5 | 2119.4-3459.4 | 2831.3 | 2455.7-3106.3 | 0.5 | 2698.5 | 2387.6-3100.8 | 2343.3 | 2073.0-2396.3 | 0.3 |
| **Potassium (mg)** | 1934.2 | 1686.8-2372.8 | 1887.0 | 1282.7-2059.3 | 0.7 | 1987.7 | 1717.3-2397.6 | 2183.0 | 1796.0-2786.3 | 0.7 |
| **Chlorine (mg)** | 4394.5 | 3250.5-4952.8 | 4640.3 | 3037.0-5071.0 | 0.5 | 2612.2 | 2489.3-3210.8 | 2540.7 | 21..03-2904.7 | 0.5 |
| **Phosphorus (mg)** | 1486.7 | 1384.7-1612.4 | 1237.0 | 1064.3-1337.3 | 0.7 | 568.2 | 439.8-768.8 | 681.7 | 598.0-884.3 | 0.3 |
| **Iron (mg)** | 8.6 | 7.0-9.5 | 8.3 | 7.1-10.0 | 0.7 | 6.2 | 4.5-6.8 | 7.3 | 5.6-7.8 | 0.2 |
| **Zinc (mg)** | 8.5 | 7.8-9.4 | 7.7 | 7.0-10.3 | 0.5 | 4.1 | 2.8-4.3 | 4.1 | 3.0-5.7 | 0.4 |
| **Copper (mg)** | 0.7 | 0.5-0.8 | 0.7 | 0.6-0.7 | 0.7 | 1.1 | 1.0-1.2 | 0.8 | 0.6-1.0 | 0.01 |
| **Manganese (mg)** | 1.3 | 1.1-2.0 | 1.8 | 1.3-2.0 | 0.3 | 2.5 | 2.3-2.7 | 2.8 | 2.1-3.6 | 0.8 |
| **Selenium (µg)** | 41.0 | 38.6-48.3 | 58.0 | 23.0-76.7 | 0.5 | 22.5 | 17.6-33.4 | 16.3 | 7.3-19.3 | 0.2 |
| **Iodine (µg)** | 198.7 | 114.5-242.4 | 126.3 | 102.0-167.3 | 0.8 | 58.7 | 41.3-98.3 | 71.0 | 50.3-96.3 | 0.5 |

**Supplementary Table 6: Faecal concentration of SCFA (µmol/g dwt) in younger(n=8) and older (n=11) participants after low and high-polyphenol diets.**

| **Group** | **Younger** | | | | | | **Older** | | | | | |
| --- | --- | --- | --- | --- | --- | --- | --- | --- | --- | --- | --- | --- |
| **Diet** | **Low polyphenol diet** | | **High polyphenol diet** | |  |  | **Low polyphenol diet** | | **High polyphenol diet** | |  |  |
| **Acid** | **Median** | **IQR**  (Q1-Q3) | **Median** | **IQR**  (Q1-Q3) | **∆** | **∆ IQR** | **Median** | **IQR**  (Q1-Q3) | **Median** | **IQR**  (Q1-Q3) | **∆** | **∆ IQR**  (Q1-Q3) |
| **Acetic acid** | 97.8§ | 76.8-103.3 | 130.2† | 108.6-134.0 | **32.4** | 4.7-42.0 | 146.7§ | 119.2-169.9 | 185.0† | 131.4-197.1 | **38.3** | 1.2-45.7 |
| **Propionic acid** | 25.6 | 16.1-35.5 | 25.9 | 20.0-33.5 | **0.3** | -14.7 | 34.5 | 19.6-40.7 | 28.4 | 20.4-29.7 | **-6.2** | -14.7 |
| **Isobutyric acid** | 3.2 | 2.4-4.6 | 2.5 | 2.1-2.9 | **-0.7** | -2.8 | 6 | 3.8-6.6 | 3.2 | 2.8-3.9 | **-2.8** | -3.6 |
| **Butyric acid** | 19.4 | 9.1-25.4 | 17.5 | 14.6-25.4 | **-1.9** | -11.1 | 28.2 | 15.7-48.5 | 26.7 | 2.3 | **10.2** | -6.4 |
| **Isovaleric acid** | 5.7 | 3.9-7.7 | 3.6 | 3.1-5.3 | **-2.1** | -3.3 | 9.3 | 8.0-11.4 | 5.3 | 5.1-6.6 | **-3.9** | -3.7 |
| **Valeric acid** | 4.9 | 2.5-6.2 | 3.3 | 2.8-4.3 | **-1.6** | -4.5 | 5 | 3.2-6.7 | 3.7 | 3.1-4.6 | **-1.2** | -5.6 |
| **Isocaproic acid** | 0 | 0.4-0.5 | 0 | 0 | **0** | -0.48 | 0 | 0 | 0 | 0 | **0** | 0.0-0.0 |
| **Caproic acid** | 1.7 | 0.9-3.3 | 1.1 | 0.0-3.9 | **-0.6** | -3.36 | 1.1 | 0.4-1.8 | 1.2 | 0.0-2.8 | **0.9** | -0.7 |
| **Enanthic acid** | 1.2 | 0.9-2.6 | 1.8† | 0.0-4.2 | **0.6** | 0.0-0.0 | 0 | 0 | 0.0† | 0 | **0** | 0.0-0.0 |
| **Caprylic acid** | 1.7 | 0.6-2.2 | 2.3 | 0.6-4.3 | **0.6** | 0.4-1.7 | 1.1 | 0.4-1.7 | 0 | 0.0-1.2 | **-0.1** | 0.0-1.2 |
| ***Total SCFA*** | ***164.3*** | ***124.4-218.9*** | ***192.4*** | ***162.1-214.9*** | ***27.1*** | ***6.5-42.7*** | ***258.4*** | ***167.9-280.3*** | ***264.5*** | ***208.3-301.0*** | ***6.1*** | ***-83.6*** |

∆ difference in faecal concentration of SCFA from low to high polyphenol diet; IQR: inter-quartile range

p values – no significant differences between groups for the Δ values

§Significant difference between groups after the low-polyphenol diet p≤0.05

†Significant difference between groups after the high-polyphenol diet p≤0.05

Supplementary Table7: Cumulated gas production (ml) from fermented faecal fluids over 24 h of incubation in A) younger (n=6) and older (n=4) participants. Data presented as median and IQR.

| **Substrates** | **Hours** |  | **Younger group** | | **Older group** | | **P value** |
| --- | --- | --- | --- | --- | --- | --- | --- |
|  |  |  | **Median** | **IQR**  (Q1-Q3) | **Median** | **IQR**  (Q1-Q3) |  |
| **Blank** | 0 | | 0 | 0 | 0 | 0 | 0.9 |
|  | 2 | | 1.5 | 0.3-4.6 | 9.5 | 7.8-9.6 |  |
|  | 4 | | 5 | 2.3-10.4 | 9.5 | 7.8-9.6 |  |
|  | 6 | | 7 | 6.3-11.1 | 9.5 | 7.8-9.6 |  |
|  | 24 | | 7.3 | 7.0-11.3 | 9.5 | 7.8-9.6 |  |
| **Raftiline** | 0 | | 0 | 0 | 0 | 0 | 0.02 |
|  | 2 | | 7.3 | 7.0-8.6 | 13.5 | 8.8-21.8 |  |
|  | 4 | | 16.8 | 12.4-20.8 | 27.5 | 24.0-36.0 |  |
|  | 6 | | 25.8 | 21.9-30.0 | 42 | 39.3-46.5 |  |
|  | 24 | | 30.5 | 28.6-32.8 | 56.5 | 46.5-63.8 |  |
| **Rutin** | 0 | | 0 | 0 | 0 | 0 | 0.2 |
|  | 2 | | 5.5 | 2.5-7.8 | 11.3 | 8.9-12.4 |  |
|  | 4 | | 12 | 9.0-13.5 | 13 | 11.4-13.8 |  |
|  | 6 | | 13.5 | 11.6-15.4 | 13 | 11.4-13.8 |  |
|  | 24 | | 18.3 | 12.6-22.4 | 13 | 11.413.8 |  |
| **Rutin & Raftiline** | 0 | | 0 | 0 | 0 | 0 |  |
|  | 2 | | 8 | 6.3-10.5 | 13.5 | 11.3-19.8 |  |
|  | 4 | | 20.5 | 14.0-21.4 | 17.5 | 14.9-27.8 | 0.4 |
|  | 6 | | 27 | 25.9-27.4 | 30 | 29.1-37.0 |  |
|  | 24 | | 37 | 30.9-39.8 | 49.5 | 33.0-67.0 |  |

**Supplementary Table 8: SCFA concentration (µmole/ml) after 0,2,4,6 and 24 h. of fermentation from younger (n=6) and older (n=4) participants.**

| **SCFA** | **Group** | **Substrates** | **0h** | | **2h** | | | **4h** | | **6h** | | **24h** | | |
| --- | --- | --- | --- | --- | --- | --- | --- | --- | --- | --- | --- | --- | --- | --- |
|  |  |  | **Median** | **IQR**  (Q1-Q3) | **Median** | | **IQR**  (Q1-Q3) | **Median** | **IQR**  (Q1-Q3) | **Median** | **IQR**  (Q1-Q3) | **Median** | **IQR**  (Q1-Q3) | |
| **C2** | **Younger** | **Blank** | 1.2 | 1.0-0.3 | 2.2 | | 2.1-2.7 | 4.1 | 3.9-4.6 | 6.0 | 5.6-6.9 | 7.8 | 7.1-9.4 | |
|  |  | **Raftiline** | 1.5 | 1.2-1.6 | 8.4 | | 7.0-9.6 | 18.1 | 14.1-19.0 | 23.1 | 20.4-24.4 | 30.7 | 26.8-34.0 | |
|  |  | **Rutin** | 1.0 | 0.8-1.2 | 2.3 | | 2.0-2.7 | 3.7 | 3.6-4.1 | 5.2 | 4.9-5.6 | 9.3 | 8.0-10.0 | |
|  |  | **Rutin+raftiline** | 1.2 | 0.8-1.7 | 6.8 | | 4.4-9.4 | 14.4 | 11.7-17.3 | 17.5 | 14.2-22.2 | 32.1 | 20.9-35.3 | |
|  | **Older** | **Blank** | 1.2 | 1.0-1.4 | 2.8 | | 2.0-3.7 | 4.9 | 4.3-5.5 | 5.8 | 5.5-6.2 | 7.8 | 7.5-7.8 | |
|  |  | **Raftiline** | 1.3 | 1.0-2.2 | 5.6 | | 3.8-9.6 | 17.7 | 14.9-20.4 | 28.1 | 26.9-29.3 | 35.8 | 31.3-42.9 | |
|  |  | **Rutin** | 1.3 | 1.0-1.7 | 2.9 | | 2.3-3.4 | 5.3 | 4.3-5.9 | 6.9 | 6.1-7.0 | 8.3 | 7.6-9.5 | |
|  |  | **Rutin+raftiline** | 1.4 | 0.9-1.9 | 6.3 | | 3.3-11.0 | 14.5 | 9.6-20.9 | 24.4 | 22.0-28.1 | 39.2 | 32.6-47.3 | |
| **C3** | **Younger** | **Blank** | 0.4 | 0.3-0.5 | 1.1 | | 0.8-1.2 | 1.7 | 1.3-2.0 | 2.2 | 1.6-2.3 | 1.8 | 1.6-2.1 | |
|  |  | **Raftiline** | 0.4 | 0.2-0.5 | 2.4 | | 1.7-3.3 | 4.2 | 3.7-4.6 | 5.3 | 4.9-7.0 | 5.7 | 4.8-9.6 | |
|  |  | **Rutin** | 0.3 | 0.2-0.4 | 1.2 | | 0.7-1.5 | 1.7 | 1.2-2.1 | 2.0 | 1.5-2.5 | 2.4 | 2.0-3.0 | |
|  |  | **Rutin+raftiline** | 0.4 | 0.2-0.5 | 2.5 | | 0.1-3.0 | 4.0 | 2.2-5.2 | 5.3 | 4.2-6.5 | 6.8 | 4.4-7.9 | |
|  | **Older** | **Blank** | 0.3 | 0.2-0.3 | 0.6 | | 0.5-0.8 | 1.1 | 1.1-1.2 | 1.6 | 1.5-1.6 | 2.2 | 1.9-2.5 | |
|  |  | **Raftiline** | 0.2 | 0.2-0.3 | 0.6 | | 0.5-1.4 | 1.5 | 1.2-2.4 | 3.1 | 2.5-3.7 | 5.2 | 4.3-6.1 | |
|  |  | **Rutin** | 0.2 | 0.2-0.3 | 0.5 | | 0.4-0.7 | 1.1 | 1.0-1.2 | 1.5 | 1.5-1.6 | 2.4 | 1.9-2.7 | |
|  |  | **Rutin+raftiline** | 0.3 | 0.3-0.4 | 0.6 | | 0.5-1.4 | 1.5 | 1.3-2.1 | 2.8 | 2.4-3.5 | 5.6 | 4.5-8.0 | |
| **iC4** | **Younger** | **Blank** | 0.1 | 0.1 | 0.1 | | 0.1 | 0.1 | 0.1 | 0.1 | 0.1-0.2 | 0.3 | 0.2-0.6 | |
|  |  | **Raftiline** | nd | nd | nd | | nd | nd | nd | nd | nd | nd | nd | |
|  |  | **Rutin** | 0.1 | 0.1 | 0.2 | | 0.2 | 0.1 | 0.1 | 0.1 | 0.1 | 0.6 | 0.4-0.7 | |
|  |  | **Rutin+raftiline** | nd | nd | 0.2 | | 0.2 | 0.1 | 0.1 | 0.1 | 0.1 | 0.1 | 0.1 | |
|  | **Older** | **Blank** | nd | nd | 0.1 | | 0.1 | 0.1 | 0.1 | 0.2 | 0.1-0.2 | 0.7 | 0.5-0.7 | |
|  |  | **Raftiline** | 0.0 | 0.0 | 0.1 | | 0.1 | 0.1 | 0.1 | 0.1 | 0.1 | 0.1 | 0.1 | |
|  |  | **Rutin** | nd | nd | 0.1 | | 0.1 | 0.1 | 0.1 | 0.2 | 0.1-0.2 | 0.7 | 0.5-0.7 | |
|  |  | **Rutin+raftiline** | nd | nd | 0.1 | | 0.1 | 0.1 | 0.1 | 0.1 | 0.1 | 0.1 | 0.1 | |
| **C4** | **Younger** | **Blank** | 0.3 | 0.2-0.3 | 0.5 | | 0.4-0.7 | 0.9 | 0.6-1.2 | 1.3 | 0.5-1.4 | 1.2 | 0.9-1.6 | |
|  |  | **Raftiline** | 0.2 | 0.1-0.3 | 1.0 | | 0.7-1.1 | 1.8 | 1.2-2.8 | 2.5 | 2.4-3.8 | 6.8 | 3.1-8.3 | |
|  |  | **Rutin** | 0.2 | 0.1-0.3 | 0.5 | | 0.3-0.5 | 0.8 | 0.6-0.9 | 1.0 | 0.9-1.1 | 1.5 | 1.2-1.7 | |
|  |  | **Rutin+raftiline** | 0.2 | 0.2-0.3 | 0.8 | 0.3-0.9 | | 1.0 | 0.5-2.2 | 1.6 | 1.5-3.1 | 3.9 | | 2.0-5.3 |
|  | **Older** | **Blank** | 0.2 | 0.2-0.3 | 0.8 | 0.5-0.9 | | 1.4 | 1.0-1.5 | 1.5 | 1.4-1.9 | 2.2 | | 2.2-2.6 |
|  |  | **Raftiline** | 0.4 | 0.2-0.4 | 1.1 | 0.8-2.7 | | 3.1 | 2.2-5.3 | 4.9 | 4.0-4.0 | 5.6 | | 5.3-12.3 |
|  |  | **Rutin** | 0.2 | 0.2-0.3 | 0.8 | 0.5-0.9 | | 1.3 | 1.0-1.4 | 1.7 | 1.4-1.9 | 2.4 | | 2.3-2.7 |
|  |  | **Rutin+raftiline** | 0.4 | 0.4-0.5 | 1.6 | 1.0-2.8 | | 4.8 | 2.9-5.6 | 7.8 | 5.2-9.4 | 7.7 | | 7.4-14.0 |
| **iC5** | **Younger** | **Blank** | 0.1 | 0.1-0.4 | 0.1 | 0.1-0.2 | | 0.2 | 0.1-0.2 | 0.2 | 0.1-0.3 | 0.8 | | 0.5-1.2 |
|  |  | **Raftiline** | 0.4 | 0.1-5.2 | 0.1 | 0.1-0.4 | | 0.1 | 0.1-0.2 | 0.1 | 0.1-0.2 | 0.1 | | 0.1 |
|  |  | **Rutin** | 0.5 | 0.3-0.7 | 0.1 | 0.01 | | 0.2 | 0.1-0.2 | 0.2 | 0.2 | 1.3 | | 0.9-1.3 |
|  |  | **Rutin+raftiline** | 0.1 | 0.1-0.2 | 0.1 | 0.1-0.3 | | 0.1 | 0.1 | 0.1 | 0.1-0.4 | 0.1 | | 0.1 |
|  | **Older** | **Blank** | 0.1 | 0.1 | 0.2 | 0.1-0.2 | | 0.3 | 0.2-0.3 | 0.4 | 0.4-0.5 | 1.2 | | 1.1-1.4 |
|  |  | **Raftiline** | 0.1 | 0.1 | 0.1 | 0.1 | | 0.2 | 0.1-0.2 | 0.2 | 0.2-0.4 | 0.2 | | 0.2-1.5 |
|  |  | **Rutin** | 0.1 | 0.1 | 0.1 | 0.1-0.2 | | 0.2 | 0.2-0.3 | 0.4 | 0.3-0.5 | 1.0 | | 0.8-1.3 |
|  |  | **Rutin+raftiline** | 0.1 | 0.1 | 0.1 | 0.1 | | 0.2 | 0.1-0.2 | 0.2 | 0.2 | 0.2 | | 0.2-0.9 |
| **C5** | **Younger** | **Blank** | 0.1 | 0.1 | 0.1 | 0.1 | | 0.2 | 0.1-0.2 | 0.4 | 0.3-0.6 | 0.5 | | 0.4-0.8 |
|  |  | **Raftiline** | 0.0 | 0.0 | 0.1 | 0.1 | | 0.2 | 0.1-0.2 | 0.2 | 0.2-0.3 | 0.2 | | 0.1-0.4 |
|  |  | **Rutin** | 0.1 | 0.1 | 0.1 | 0.1 | | 0.1 | 0.1 | 0.2 | 0.2-0.3 | 0.9 | | 0.8-1.0 |
|  |  | **Rutin+raftiline** | 0.0 | 0.0 | 0.1 | 0.1-0.2 | | 0.1 | 0.1 | 0.1 | 0.1-0.2 | 0.1 | | 0.1-0.2 |
|  | **Older** | **Blank** | nd | nd | 0.3 | 0.2-0.3 | | 0.8 | 0.8 | 0.5 | 0.1-0.9 | 1.1 | | 1.1 |
|  |  | **Raftiline** | 0.0 | 0.0 | 0.3 | 0.3 | | 0.5 | 0.3-0.5 | 0.6 | 0.4-0.6 | 0.6 | | 0.4-0.6 |
|  |  | **Rutin** | nd | nd | 0.2 | 0.2-0.3 | | 0.7 | 0.7 | 0.9 | 0.5-0.9 | 1.0 | | 1.0 |
|  |  | **Rutin+raftiline** | 0.1 | 0.1 | 0.3 | 0.3 | | 0.5 | 0.5 | 0.6 | 0.3-0.7 | 0.6 | | 0.4-0.7 |

Not detected (nd), acetic acid (C2), propionic acid (C3), isobutyric acid (iC4), butyric acid (C4), isovaleric acid (iC5), valeric acid (C5)

**Supplementary Fig 1. Change in faecal pH after low and high-polyphenol diets in the younger (n=8) and older (n=10) participants.**

Each circle indicates the measurement of faecal pH for each participant after LPD and HPD. Median faecal pH for each group is indicated by a red horizontal line.

a,b symbols indicate differences within group (LPD to HPD).

Supplementary Fig 2. Faecal SCFA (µmoles/g dwt) after low and high-polyphenol diets in younger (n=8) and older (n=11) participants.

Each circle indicates the measurement of faecal SCFA for each participant after LPD and HPD. Median faecal SCFA for each group is indicated by a red horizontal line.

§ symbol indicates differences between groups (high vs. high).

Supplementary Fig 3. Change in pH of fermented faecal fluids over 24 h in A) younger (n=6) and older (n=4) participants. Data presented as median and IQR.

**(A)**


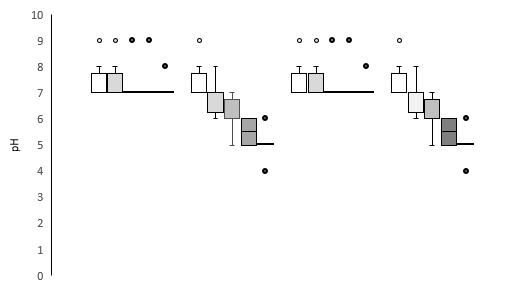


**Blank**

**Rutin**

**Rutin & Raftiline**

**Raftiline**

**(B)**


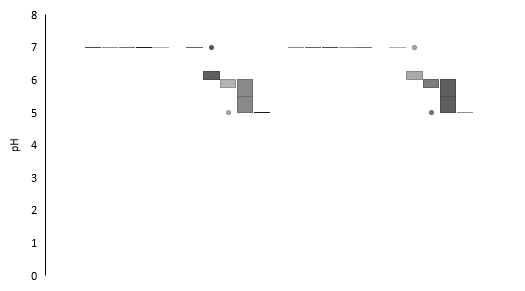


**Blank**

**Rutin & Raftiline**

**Raftiline**

**Rutin**

pH data is presented for each time point (t=0, 2, 4, 6, 24hr, in a colour gradient). When all data points are the same, the median value appears as a single line without box or whiskers.
